# Progress in Fevers

**Published:** 1904-01-23

**Authors:** 


### PROGRESS IN FEVERS.

*(Concluded from page 275.)*

Malaria.—Under the head of "hybrid malaria" J. L. Porteous[16] describes an outbreak of fever occurring in Yonkers, New York. The chief features were a remittent continued fever with clusters of discrete rose-coloured spots, not however coming out in crops, muscular pain, insomnia, and sometimes diarrhœa and tympanites. The onset is more sudden than in the case of typhoid fever, and begins with chilliness of the extremities and flushing of the face. The duration is one to four weeks. The Widal reaction was not obtained. The cases showed some features of malaria and many of typhoid, but were not considered to be due to typhoid or para-typhoid infection. Warbury's tincture proved a useful remedy, one case of diarrhœa did well with acetozone. E. L. Waters,[17] I.M.S., describes malaria as observed by him in the Andamans Penal Settlement. The incidence of the disease in this settlement is very heavy, nearly 14,000 admissions for a strength of about 11,000 in 1902, and although actual deaths from this cause numbered only 57, the lowered vitality induced by malaria probably contributed to many deaths from other causes such as dysentery and tuberculosis. What the writer calls the purely mosquito theory of infection does not in this region fully account, in his view for the incidence of malaria. Recourse must also be had, he thinks, to the relapse or recrudescence theory. Practically all the natives who came to the Andamans have previously suffered from malaria, and the disease is apt to reappear in those who are run down from overwork, exposure or chill. Reference is made to the writing of Caccini, of Rome, who has found malaria reappear within 48 hours of exposure to some debilitating cause. Such a recrudesence may take place, he says, after 12 months of freedom from fever. With regard to the measures adopted for diminishing malaria, the three standard recommendations have all been carried out more or less thoroughly, viz., destruction of mosquitoes, the use of mosquito curtains, and the prophylactic dosing with quinine. Also it is regarded as very important to keep the population as "fit" as possible with a view to avoiding relapse. Destruction of mosquitoes and their larvæ has been aimed at and carried out successfully in those parts of the settlement where this was feasible, but with disappointing results as far as diminished incidence of malaria was concerned. Sleeping under mosquito nets was found effective. In the female jail where it was tried malaria was reduced to one-fourth. The great drawback, however, is its interference with ventilation—a very serious matter where 20 or 30 persons must sleep under one large net. The general use of nets would require a very considerable increase of cubic space per head. The use of quinine as a prophylactic did some good, but was of less value than theoretically might have been expected. One of the commonest parasites found, however, in the settlement, is the malignant crescent, and this form, it is well known, is particularly resistent to quinine. For treatment in some of these obstinate cases methylene blue sometimes does well. Lately "mass doses" of quinine, 30 grs. by the mouth or hypodermically have been tried with success. Hæmoglobinuria is unknown. In Ismailia[18] the prophylactic measures recommended by Ross have been carried out with most satisfactory results. Malaria has greatly diminished, and of the cases still found many are regarded as examples of relapse. A. R. Waddell[19] has experimented on mosquito larvæ, and finds that these are destroyed in very dilute solutions of ammonia, such as 1 in 20,000 or 1 in 30,000. Arguing from this fact, he thinks that mosquito growth might be usefully inhibited by nitrification of the soil and of many surface waters. Agricultural chemists know that certain leguminous plants, by their peculiar root action, add nitrogen extracted from the air to the soil in which they live. This nitrifying action is brought about by certain earth bacteria which are found about the roots of the leguminosæ. The bacteria are abundant in good soils containing plenty of phosphates, potash, and lime. They are also sold commercially under the name of "nitragin." Waddell suggests that advantage of these facts might be taken in cultivating lands which breed mosquitoes by applying phosphatic manures and planting the land with leguminosæ. A. D. E. Kennard[20] draws attention to the usefulness of sodium salicylate in malaria and its superiority to quinine in reducing fever and relieving pain. Three cases are quoted, all of which occurred on board ship, where an examination of the blood was not possible. Two patients had severe pains in the thighs and legs, and one had pain, tenderness, redness, and swelling of both ankles. The methods of Manson and Ross[21] differ somewhat in detail in dosing with quinine. Manson's doses are smaller and less frequent, and he advises drinking freely, for four or five months, of lemonade, made by boiling in water the whole of a lemon cut into small pieces. A calomel purge is given occasionally. Both authorities advise that quinine should be gradually decreased after an attack of malaria, but that it should be regularly continued in decreasing doses for at least four months.

[16] Med. Rec., July 25. [17] Lancet, June 13 [18] Brit. Med Jour., Aug. 22, p. 427. [19] Lancet, June 6. [20] Lancet, July 11. [21] Med. Rec.. July 4, p. 36.

